# Psychosocial support for adolescent girls in post-conflict settings: beyond a health systems approach

**DOI:** 10.1093/heapol/czx127

**Published:** 2017-12-09

**Authors:** Fiona Samuels, Nicola Jones, Bassam Abu Hamad

**Affiliations:** 1 Social Development Programme, Overseas Development Institute, 203 Blackfriars Road, London SE1 8NJ, UK; 2 Faculty of Public Health, Al-Quds University, 101, Tel El Hawa, Gaza, Palestine

**Keywords:** Psychosocial, adolescence, girls, post-conflict, social determinants of health, wellbeing, health, Gaza, Liberia, Sri Lanka

## Abstract

Adaptive and adequately resourced health systems are necessary to achieve good health outcomes in post-conflict settings, however domains beyond the health system are also critical to ensure broader wellbeing. This paper focuses on the importance of psychosocial support services for adolescent girls in fragile contexts. Its starting point is that adolescence is a pivotal time in the life course but given the physical, cognitive and emotional changes triggered by the onset of puberty, it can also be a period of heightened sensitivity and vulnerability to trauma, social isolation, bullying by peers, a lack of supportive adults and gender-based and sexual violence. Our findings highlight why humanitarian and biomedical approaches in their current form are inadequate to address these complexities. Drawing on qualitative fieldwork (consisting of in-depth and key informant interviews as well as group discussions in Gaza, Liberia and Sri Lanka involving a total of 386 respondents across the three countries), we argue that going beyond biomedical approaches and considering the social determinants of health, including approaches to tackle discriminatory gendered norms and barriers to service access, are critical for achieving broader health and wellbeing. While all three case study countries are classified as post-conflict, the political economy dynamics vary with associated implications for experiences of psychosocial vulnerabilities and the service environment. The study concludes by reflecting on actions to address psychosocial vulnerabilities facing adolescent girls. These include: tailoring services to ensure gender and age-sensitivity; investing in capacity building of service providers to promote service uptake; and enhancing strategies to regulate and coordinate actors providing mental health and psychosocial support services.

Key Messages

- Adaptive and adequately resourced health systems are necessary but not sufficient to ensure good health outcomes among adolescent girls in post-conflict settings.
- Integrating an understanding of the social determinants of health and wellbeing is critical in shaping health service uptake and ultimately outcomes for adolescent girls.
- Psychosocial outcomes are also central to broader health and wellbeing and need urgent attention in post-conflict developing country settings in particular.
- Tailored gender- and age-sensitive psychosocial service provisioning is vital in supporting the health and wellbeing of adolescent girls—a cohort often facing complex vulnerabilities in post-conflict settings.

## Introduction

Adaptive and adequately resourced health systems are necessary to ensure good health outcomes in fragile and post-conflict settings. Nevertheless, it is increasingly recognised that domains beyond the health system also significantly impact on broader health and wellbeing. This paper focuses on the importance of psychosocial services for vulnerable populations and specifically adolescent girls in fragile contexts. Adolescence is a pivotal time in the life course when there is considerable opportunity for change. At the same time it is also a period when adolescent girls are especially vulnerable to a range of life-changing experiences including trauma, social isolation, bullying by peers, a lack of supportive adults, and sexual and other forms of violence. The aims of the study are to highlight why current humanitarian and biomedical approaches in the current form are inadequate to address these complexities. It also stresses how critical an understanding of the social determinants of health is to enable adequate and context appropriate responses.

This paper argues that it is important to go beyond biomedical approaches and instead investigate the complex social determinants of health critical for achieving broader health and wellbeing, especially amongst the most vulnerable. There is now more evidence that explores the links between gender and adolescence, mental health and development (e.g. Priess et al. 2009; Stavropoulou and Samuels 2015). Yet in many developing countries, health system debates and research have tended to overlook mental health and psychosocial wellbeing generally (e.g. Bragin 2014) and particularly the needs of adolescent girls in post-conflict or other fragile settings (for further details of relevant literature see Stavropoulou and Samuels, 2015). To contribute to these debates, we draw on qualitative fieldwork from 2014 to 2015 in three countries recently affected by conflict—Gaza, Liberia and Sri Lanka—honing in on the psychosocial vulnerabilities of girls aged 10–19 and the extent to which these are addressed by the existing psychosocial service environment.

## Situating our study within the wider literature

Drawing on a large and diverse body of literature, in this section we discuss the conceptual underpinnings of this study, making explicit linkages between the social determinants of health and the mental health and psychosocial wellbeing of adolescent girls. According to the World Health Organization (WHO) ‘The social determinants of health (SDH) are the conditions in which people are born, grow, work, live, and age, and the wider set of forces and systems shaping the conditions of daily life. These forces and systems include economic policies and systems, development agendas, social norms, social policies and political systems’^1^. The ways in which these conditions are experienced by those at risk of psychosocial ill-being are refracted through a number of individual level variables, including age and gender, and family and community level factors, including discriminatory gendered social norms (see e.g. Harper et al. forthcoming).

### Why the focus on adolescents, particularly girls?

Although definitions vary, the United Nations defines adolescence as the second decade of life between 10 and 19 years (UNICEF 2011). During this time, girls and boys undergo bodily changes (e.g. the start of menstruation, the development of breasts for girls and deepening voice and hair growth for boys) and begin the transition from childhood to adulthood, when they will assume roles such as parent, spouse, worker and citizen. During adolescence, the constraining role and influence of gendered social norms on girls’ lives (in a range of domains, from education and marriage to mobility outside the home) becomes more evident (Jones et al. 2010; UNICEF 2011). It is also during this period that developmental changes occur and key skills are acquired, such as those that relate to: health, physical and neurological development; social behaviours and attitudes; and education and employment. For girls and young women in many settings, this life stage also heightens deprivation, danger and vulnerability, including an increased risk of sexual and domestic violence. This constrains girls’ ability to develop agency and leads to other development deficits, often leading to negative consequences in their adult lives (see e.g. Hallman et al. 2015 and Gage 2013). Other context-specific factors, such as national laws and local cultural norms and practices also have a major impact on adolescent girls’ lives, dictating not only how girls should think and behave but how others expect girls to behave.

In this study, the national context (of a fragile or post-conflict state) shapes how girls and their families recover from or continue to face violence, disruption, displacement and loss of livelihoods. These experience are often gendered, as social norms shape how families react to and cope with external shocks and stresses, often with negative outcomes for girls including marrying them off early and pulling them out of school (e.g. Mazurana et al. 2013).

### From mental health to psychosocial wellbeing

Mental health is now recognised as being crucial to overall health (e.g. WHO 2001) and is defined as including ’… subjective well-being, perceived self-efficacy, autonomy, competence, intergenerational dependence, and self-actualization of one’s intellectual and emotional potential, among others’ (WHO 2001, p. 5). Alongside the suffering it causes, mental ill-health can exacerbate household poverty, increase inequality, reduce social capital, and hinder economic growth (WHO 2010). Being exposed to situations common to post-conflict contexts, such as violence, death and difficult living conditions (e.g. lack of food, epidemics, or being forced to flee one’s home) is likely to negatively affect the psychosocial wellbeing of individuals and families (see e.g. Betancourt et al. 2014; De Jong, 2002).

Sexual violence (largely directed against women and girls), a known risk factor for mental ill-health, is also heightened in conflict contexts. Even after a conflict has ended, girls and women often continue to be subject to sexual and other violence. This often reflects a certain ‘normalisation’ of such violence as well as continuing availability of weapons, lack of punishment for perpetrators, and frustration among men who are no longer able to live up to the role expected of them by traditional norms (see e.g. Baksh et al. 2005 and Domingo et al. 2013).

### Mental health and psychosocial risks in adolescence

Mental ill-health and psychosocial problems typically start during adolescence; unless young girls or boys receive appropriate treatment, the results of these psychosocial problems will continue to affect them as adults (WHO 2007). One study noted that between 10% and 20% of children and young people have been reported as experiencing diagnosable mental illnesses (Kieling et al. 2011). This is leading to rising suicide rates, with young people now the highest-risk group for suicide in around 30% of all countries (WHO 2007, 2016); in China, India and the South East Asia region, suicide is the leading cause of death among those aged 15–19 (Patel et al. 2007; WHO 2014).

Other negative consequences of mental ill-health and psychosocial problems for young people include poorer school attainment, resorting to substance abuse, greater likelihood of experiencing violence, and increased likelihood of facing reproductive and sexual health-related challenges (Patel et al. 2007; Kieling et al. 2011). Despite widespread and increasing awareness of the mental health challenges facing adolescents, their needs in this area are largely unmet, and particularly in developing countries. Reasons for this relative neglect include lack of effective policies targeting adolescents, limited funding and human resources (particularly qualified healthcare professionals), limited training and experience of lower-level health staff, and the ongoing stigma linked to mental ill-health (ibid.).

Adolescence is recognised as a troubling time in a person’s development, but it brings particular challenges for children and young people in conflict/post-conflict situations. They may experience traumatic events such as the death of (or separation from) their parents; they may be subject to sexual and/or physical violence; they may be abused, abducted, or forced to become involved with fighting forces; and they may miss out on their schooling and on other social and economic opportunities (Boyden and de Berry 2004). All these experiences can bring on serious mental ill-health and emotional trauma, and can have negative and long-term impacts on how adolescents see themselves, interact with each other as well as on their aspirations for the future. These experiences are often gendered—in a study of Sierra Leone’s child soldiers Betancourt et al. (2011), for instance, found that girls demonstrated significantly higher scores of depression and anxiety than boys (80% vs 52% and 72% vs 55%, respectively), partly explained by the fact that girls experienced many more cases of rape and sexual abuse than boys (44% vs 5%).

### Towards a norms-sensitive conceptual framework for understanding psychosocial wellbeing

Humanitarian actors increasingly recognise the impact of conflict and disaster on people’s psychosocial wellbeing and in recent years have incorporated mental health and psychosocial support (MHPSS) into their response (Bragin 2014; Ward and Marsh 2006; Wessells 2008). The 2004 Indian Ocean tsunami was arguably a turning point, as it required a systematic response to people’s mental health as well as physical survival needs, with a focus on building individual and community resilience (UNHCR 2013; Stavropoulou and Samuels 2015).

This has also given rise to new conceptual frameworks that focus on different resource domains that are affected in situations of conflict. These include: human capacity (individuals’ mental and physical health, skills and knowledge); social ecology (the relationships that allow people to exist as a community, sometimes called social capital); material and physical environments; and local values and culture (Strang and Ager 2003; Galappatti 2003; White 2009). Building on these psychosocial wellbeing frameworks, we apply a social norms lens, positing that social norms affect all domains of adolescents’ lives, not just education and marriage but also access to resources (economic, material and human), their health and wellbeing, and their ability to participate in community life (agency and voice) (Samuels et al. 2015). Applying a social norms lens helps us to understand and put in context the different paths to wellbeing that are available to adolescents, as well as the challenges faced by girls (and boys), and what works to address or overcome these challenges and barriers.

**Figure 1.**
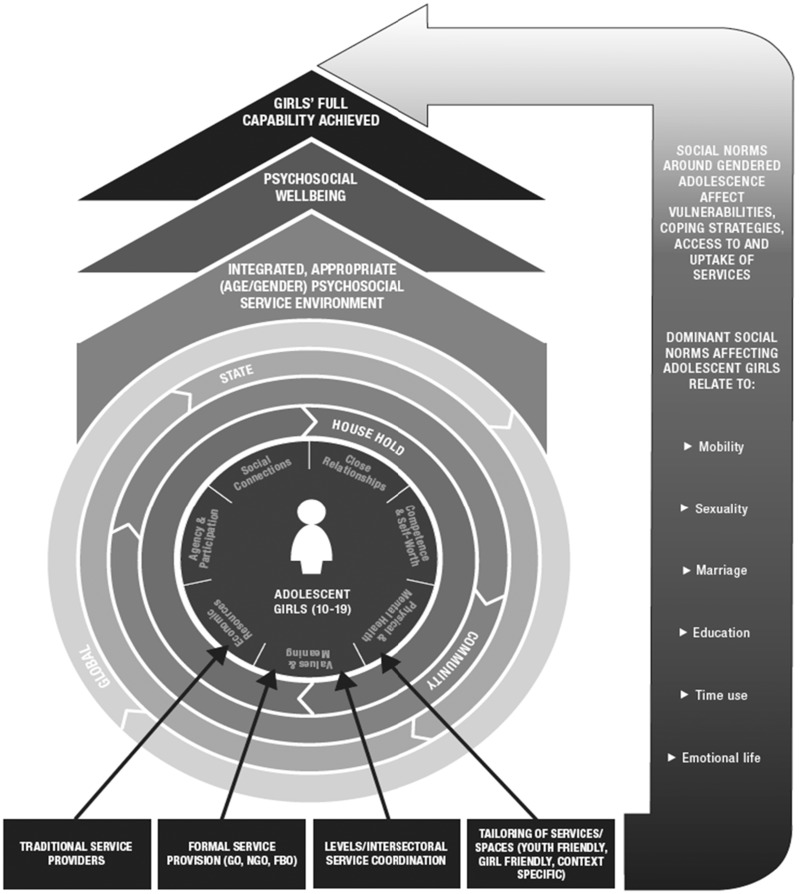
Pathways towards adolescent girls’ psychosocial and broader wellbeing

As seen in Figure 1, this approach puts adolescent girls centre stage, with key areas of wellbeing around them. Girls are situated within their families/households, their wider community and the (fragile) state, characterised by ongoing or recent conflict or other crises related to health, food security or natural disasters. These different areas of girls’ lives are in turn influenced by changes in the global context, which include greater learning around adolescents’ needs and the rise of MHPSS programming.

The availability of local services also affects girls’ psychosocial wellbeing, with tailored services being especially critical to support their evolving needs (e.g. around menstruation, information on family planning, etc.). Such service provisioning needs to be informed by an understanding of the social determinants of health (e.g. gender, ethnicity and fragility) and the importance of integrating a mix of formal, faith-based providers, and informal services. Prevailing gender norms (around education, marriage, sexuality and mobility) also determine girls’ vulnerabilities, their access to and uptake of relevant services, and their ability to develop their full capabilities. Services must be carefully targeted and designed to meet girls’ needs if they are to maximise girls’ psychosocial wellbeing and allow them to develop to their full potential.

## Methods and case study contexts

The study design combined primary qualitative data collection with a secondary review of literature. This allowed us to situate our research within broader debates on adolescence, fragile states, and mental health and psychosocial services.^2^

The research involved the collection of primary qualitative data, in conjunction with national study partners, in Sri Lanka, Liberia and Gaza in 2014 and 2015. The full research proposal and instruments, which were developed jointly with the national partners, were submitted to and approved by the Overseas Development Institute’s ethics committee. The study was also cleared by national research ethics boards in the study countries, i.e. in Liberia the Carter Centre has its own ethics board, in Gaza the study was reviewed by the Helsinki Framework ethics board, and in Sri Lanka the study partners made the necessary arrangements to conduct the study.

Phase one of the data collection aimed to map people’s perceptions (adults and young people) of the different dimensions of wellbeing (especially those affecting girls) and the factors that affect their wellbeing. The next phase focused on the availability of services, particularly looking at whether MHPSS programmes were sensitive to adolescents’ (and especially girls’) needs and how services could be made more responsive to girls’ needs.^3^Table 1 provides an overview of the study contexts and Box 1 provides an overview of the methodology.

**Table 1.** Study sites and relevant country contexts

Topic	Gaza	Liberia	Sri Lanka
Study sites	Shajaia neighbourhood *Gaza City*: high level of violence by Israeli forces leading to high numbers of IDPs: out of a total population on 120 000, 25% are refugees (NECC 2014). High rate of unemployment: >50% of the residents receive social assistance (Ibid.). Conservative community: median marriage age for women is under 20 years old (Abu Hamad et al. 2015)	New Kru town *Montserrado county*: densely populated borough of capital city, Monrovia (∼70 000 people) (Samuels et al. 2015). One of the communities that suffered most from Ebola virus and the civil war (Montserrado acted as an entry point for rebel armies entering Monrovia) (Ibid.).	Diyagama village *Polonnaruwa District, North Central Province*: Majority of population (total of 7000) belongs Tamil ethnic group (Robinson and Ross 2007). Until 2006 the area served as a Liberation Tigers of Tamil Eelam stronghold (Emmanuel et al. 2015).
			Kadalkiraman *Batticaloa District, Eastern Province*: highest level of poverty in the country (poverty head count index of 20.3%) and highest poverty gap index (5.1 compared with a national of 1.7) (UNDP 2012). Majority of the population (total of 2464 are Tamil (Emmanuel et al. 2015). Located in high-security area with large military camps. Good road: easy access to services (Ibid.).
		Tubmanburg *Bomi county*, is a city of 13 114 people in one of the poorest counties in Liberia (Bockstael and Vlassenroot 2011). During the civil war Tubmanburg served as rebel army LURD’s headquarters. In total Bomi country authorities reported 175 Ebola related deaths since the Liberian outbreak started in 2014 (MoHSW 2015).	
Population, including youth	Population: 1.71 million (1.2 are refugees): nearly half the population is under the age of 15 (PCBS 2012a). Ethnicity: 99% are Palestinian Arabs, 1% other (Ibid.).Religion: Predominantly Muslim: 99.3%, Christian 0.7% (Ibid.).	Population: 4.19 million with nearly half under 15 and 20.4% between 10 and 17 years old (World Bank 2015a, 2015b). Ethnicity: 2.5% Congo people (descendants of former slaves) and 95% indigenous (Samuels et al. 2015). Religion: 40% Christian, 20% Muslims and 40% indigenous beliefs (Ibid.).	Population: 20.3 million with 23.2% of the population between 15 and 29 years (UNDP 2014). Ethnicity: The majority is Sinhalese and 16.5% are Tamil (UNDP 2012). Religion: majority are Buddhist and 9.3% Muslim (Ibid.).
Fragility context—economic situation and conflict legacy	Fragility: Gaza is marked as a ‘hostile entity’ by Israel—characterised by blockades (since 2007 and ongoing) and military operations (MoH 2014). Economy: Growth slowed from 6% in 2012 to 2% in 2013 (in Palestine) (World Bank 2015a, 2015b). Gaza has an HDI of 0.736 (UNDP 2015). Conflict legacy: Conflict-related chronic stress has negatively impacted the public health of the population of Gaza and has, amongst other things, caused high levels of non-communicable diseases including mental health and psychosocial challenges (Abu Hamad et al. 2015).	Fragility: Civil conflict (1989–2003) due to ethnic tension causing 150.000 deaths and displaced 850.000 Liberians (UN 2015). Ebola outbreak 2014–2015 (WHO 2015). Economy: Growth rate of 7.5% in 2014. HDI of 0.412 in 2013 (UNDP 2014).	Fragility: Violent uprisings between 198 and -2009 caused by socioeconomic and ethnic grievances by Tamils. Country was hit by a tsunami in 2004 killing 35 000 people (Emmanuel et al. 2015).
			Economy: Gained middle income status in 2010 (Ibid.). HDI increased by 28% between 1980 and 2012 (HDI in 2012: 0.715). Human development indicators lagging in several provinces and rural areas (UNDP 2014).
		Conflict legacy: 48% of the population living in extreme poverty. High unemployment, food insecurity (UN Liberia 2013).	
			Conflict legacy: Conflict caused loss of thousand lives (likely as many as 330.000 deaths) injuries and disability (∼40.000 surgical procedures and 5.000 amputations during the conflict), destruction and displacement (290.000 Sri Lankans displaced from the battle zone) (UN 2011).
Gendered effects of conflict including VAWG	Gendered vulnerabilities: Conflict limits women’s already low mobility and restricts their agency and ability to build up social networks (Abu Hamad et al. 2015).Many women are displaced and live in temporary mixed housing arrangements that are not constructed gender-sensitively (mixed living room areas and lack of adequate toilet facilities) (Ibid.).	Gendered vulnerabilities: Higher rates of illiteracy for women (adult literacy rates: 37% for girls and 63% for boys (UNICEF 2007). With only 58.2% of all women participating in the labour force, most are forced to engage in vulnerable employment (UNDP 2013). Violence: During the civil conflict between 61% and 77% of all women were raped. Normalisation of GBV including FGM/C, early marriage and polygamy, sexual and domestic violence (UN 2013). Over 34% of women reported domestic violence by their partners (Greenberg 2009).	Gendered vulnerabilities: Conflict restricted movement of women and girls impacting negatively on their education and livelihoods (IGC 2013). Conflict changed traditional gender norms: in some cases girls and women became combatants or were responsible for household livelihoods, leading to a form of female empowerment (Somasundaram 2003)
			Violence: Within a context of conflict, brutalisation and militarisation opportunities for sexual violence have been normalised for women (Ibid.).
	Violence: High level of domestic violence: 51% of married women experienced violence at the hands of her husband (PCBS 2012b). Girls in temporary shelter arrangements are extra vulnerable for gender based violence and sexual abuse (Abu Hamad et al. 2015).		
Mental health policy and services context	Policy: National Health Strategy (2012–2016), Public Health Policy for Children (2012) Service providers: Ministry of Health established 54 Primary Health Care centres and 13 hospitals, but only one psychiatric hospital. Other providers include: UNRWA who established 22 centres that provide mental health services for refugees; NGOs that are providing mostly costly secondary and tertiary services in > 50 clinics; Private-for-profit operators that are mainly focussed on obstetrics and surgical intervention (MoH 2014).	Policy: National Mental Health Policy (2009), Basic Package of Health Services (2010).	Policy: National Mental Health Policy (2005–2015).
			Service providers: Policy established by the National Institute for Mental Health: national strategy aims to reduce stigma and discrimination and calls for mental health legislation (Emmanuel et al. 2015). Government provided mental health training for primary health care doctors (WHO 2013). GBV Desks at hospitals intervene in rape and sexual abuses cases and provide counselling to survivors (Emmanuel et al. 2015). Child protection officers raise awareness on the importance of child protection and offer mental health services for children (Emmanuel et al. 2015).
		Service providers: Only one hospital (private—with only one trained psychiatrist) in Liberia providing mental health services: Grant Memorial Mental Hospital in Monrovia. Primary health personnel lack training. Other mental health services are provided by NGOs or FBOs. Health systems were further damaged by the Ebola outbreak (Samuels et al. 2015).

Box 1. Methodology

We used a range of qualitative tools including: in-depth interview (IDI) guides for adolescent girls, boys and parents; key informant interview (KII) guides for service providers and community leaders; focus group discussion (FGD) guides with parents of adolescents and service providers; and guides for carrying out intergenerational trios–where a grandmother/grandfather, mother/father and daughter/son are interviewed individually to obtain information about stasis and change across the generations (for further information see: http://www.odi.org/sites/odi.org.uk/files/odi-assets/publications-opinion-files/9826.pdf). Drawing on our conceptual framework, these tools addressed sets of questions around different wellbeing domains and different levels of the social ecology. The aim was not to explore psychometric measures or properties but rather to understand the ways in which different dimensions of adolescent girls’ wellbeing are shaped by household, community, national and global factors and contexts.

To encourage respondent participation, we also integrated visual techniques into FGDs, including community timelines and mapping exercises. These techniques helped us to gain insights into what services for adolescents exist in the focal communities, changes over time in service provision and their effects. In all countries, a mapping of mental health and psychosocial services was also carried out alongside a validated Health Facility Assessment tool to assess the appropriateness of the mental health and psychosocial services provided at facilities and to identify gaps in human resources, institutional infrastructure, and other resources.

A total of 386 respondents participated in the study across the three countries (see table below). The relatively large sample size was determined by a combination of resource parameters, the importance of triangulating findings across diverse informants as well as the principle of research saturation, i.e. reaching a point where no new insights were being garnered by additional interviews.

**Table:** **Type of respondent, by tool and country**

Type of respondent	Gaza	Liberia	Sri Lanka	Totals
	(1 round of data collection)	(2 rounds of data collection)	(2 rounds of data collection)	
Adolescents	6 FGDs (44 participants in total—30 girls, 14 boys) 12 IDIs (8 girls, 4 boys)	27 IDIs (18 girls, 9 boys) 9 FGDs (5 participants in each)	64 IDIs (22 girls, 38 boys)	192
Adults—parents/caregivers, key informants	27 IDIs/KIIs	12 IDIs/KIIs 4 FGDS (involving 14 mixed sex adults/community leaders)	76 IDIs/KIIs	171
Service providers	1 FGD (6 participants)	7 IDIs/KIIs	2 FGDs (5 participants in each)	23
Total respondents	89	147	150	386
Facility assessments	5	3	5	13

In each country local researchers with expertise in mental health and psychosocial wellbeing worked alongside an international researcher. Following an initial 4 day training (which included role plays, discussions on how to deal with sensitive issues, piloting and revision of instruments), the teams spent ∼2 weeks in the field gathering data. The identification and selection of respondents in all countries was guided by local contacts, key stakeholders (e.g. service providers) and NGOs working in the study sites. Following initial interviews, snowball sampling was also used. Table 1 provides details of the study sites in each country.

After obtaining informed consent from research participants, interviews were recorded, transcribed and translated by in-country research partners. A detailed thematic coding structure was developed jointly by the international and country teams and findings from the interviews were analysed following this set of codes and sub-codes. In terms of quality control, daily debriefing sessions were held to facilitate ongoing analysis and reflection, and a subset of the transcripts (both in local languages and translated) were also read by the in-country study leads to ensure quality and consistency. We also had an external peer reviewer who provided detailed feedback on design and analysis questions at each stage of the study. Finally, in-country consultative meetings were organised with key stakeholders, to share and further examine findings comparing these with existing evidence and tacit knowledge, allowing also for the emergence of alternative interpretations.

Limitations of the study included the absence of baseline data prior to conflict episodes and resulting challenges in teasing out conflict-specific drivers shaping the outcomes of the mental health and psychosocial services provided to adolescent girls. Additionally, it was challenging to recruit mental health service users—as originally intended in the study—because of confidentiality requirements and their psychological status. As such, the study teams interviewed both adolescent service users and non-service users and was able to obtain sufficient insights from them, as well as from other respondent (especially parents and service providers), about their perceptions of their own and others’ mental health and psychosocial status. Given that we emphasise the importance of context specificity, different research teams gave different emphasis to the study components as appropriate to their settings. For example, in Liberia there was a greater focus on mental health and psychosocial support for sexual violence survivors which is a major part of the post-conflict landscape, whereas in Sri Lanka the team placed greater emphasis on issues of stigma surrounding adolescent experiences and the deeply entrenched gendered social norms associated with pubertal development which permeate both service provider attitudes and uptake of services.

## Results

### Understanding adolescent psychosocial vulnerabilities in fragile contexts

In this section we explore how adolescents in our case study countries perceive the factors underpinning their psychosocial health and the risks to it, linking to a number of key domains mapped in Figure 1.

#### Economic resources

Most of the girls in our sample identified access to economic resources (including education and livelihood opportunities) as key to their psychosocial wellbeing. In Sri Lanka, study respondents noted that while adolescent girls and their parents strongly valued education, many girls still dropped out of school due to poverty, romantic relationships or early marriage (‘*Definitely, they won’t come back to school when they elope’*, 14-year old girl, Diyagama). In Liberia, all our study respondents (girls and their parents/carers) noted that education was vital to young people’s future, in spite of substantial challenges around supply (e.g. quality of schooling and educational infrastructure) and demand (e.g. financial constraints):

‘. my mother used to advise me… not to go around with boys, that once I had a good education, no man would be able to bluff me’ (FGD, adolescent girls, New Kru Town).

In all countries, respondents reported that limited access to economic assets and livelihood opportunities by their parents/carers also affected girls’ wellbeing. In Liberia, in particular, girls spoke about this causing both themselves and their parents/carers high levels of stress. It also led them to engage in risky sexual behaviours,^4^ as this quote highlights:

‘We engage in sexual activities because at times, the things we wish to have our parents are not able to afford them; … At times our parents coerce us to get involved in early sex…saying don’t you see your friends going out there’ (adolescent girl in FGD, New Kru Town).

#### Social connections and close relationships

Supportive and caring relationships with family and others played a pivotal role in the psychosocial wellbeing of adolescent girls in our sample. In Sri Lanka, most girls reported one or both parents as their closest relative, providing the most day-to-day support.

‘I tell everything to amma (mother). I tell amma first and then after the others’ (girl, 16, Kadalkiramam).

However, many girls also reported feeling disappointed or frustrated by their parents—for instance, citing ‘a father’s drunken behaviour’ or ‘being punished by a parent’. Some girls in our sample were living with an older relative (usually female) either because the family had split or one of her parents had died. While some girls described these relationships as loving and positive, others were treated badly and even traumatised by the actions of these relatives. Girls in Sri Lanka reported that relationships beyond the family—with teachers, neighbours and peers, for example—were also important, especially for obtaining advice, sharing problems and having fun.

In Liberia, many girl respondents were not living with their biological families, which was a source of considerable pain and anxiety. Even so, they noted that relationships with family (whether immediate or extended), peers and neighbours were vital to their psychosocial wellbeing.

‘we also learn from our friends; we learn from other people; how they behave; we learn the good things from our friends not the bad things’ (girl, 17, Bomi).

In Gaza, the role of family support was also mixed. While some girl respondents sought advice and support from family members, others were more reluctant, afraid that their parents might not listen or may inadvertently make the problem worse:

‘If I share the problem with my mother, the problem could become bigger because I know mom will inform my dad of it’ (girl, 14, Gaza).

These problems were also observed by specialists working with adolescents, as the following quote shows:

‘*Adolescents feel that they are not adequately valued by the family and the community. Families don’t understand the needs of adolescents. There are many communication gaps between adolescents and their families: adolescents don’t understand parents’ concerns and worries about them and also parents don’t understand needs, aspirations and desires that adolescents have. It’s a mutual misunderstanding’ (social protection specialist, Gaza City).*

#### Competence and self-worth or self-esteem

Our findings were mixed regarding self-worth and self-esteem. Despite strong cultural and economic constraints, a significant number of girls in Liberia and Sri Lanka demonstrated a strong sense of self-esteem, describing ambitious plans for the future:

‘I wish to study well and be a teacher, build a house and look after my parents’ (girl, 14, Diyagama, Sri Lanka).

‘I want to own house. I want my own money. I want to be working for myself’ (girl, 14, Bomi, Liberia).

Study respondents—girls, their parents, and service providers –cited certain factors that were indicative of good self-esteem, including: having the love and support of parents and other relatives; having space to take part in events at school or in the community; and having hopes for a better future. Obstacles to positive self-esteem included: living in a remote area and lack of opportunities for young people to use their skills and talents; lack of places where girls are allowed to meet and socialise; and negative attitudes towards adolescents and young people.

In Gaza, however, girls repeatedly complained that their family and community did not value them equally with boys, particularly when it came to sharing their views and thoughts. One girl aged 15 said: ‘*My grandfather, when we want to speak, says*, *“You shut up, what do you think you know? You keep yourself out of things that aren’t your business.*”’

#### Physical and mental health

A theme common to all three countries (notably Liberia) was the sense of inadequate protection and security, which mapped closely onto the physical and mental health domain. In Liberia, difficult family life and abuse of girls (physical and sexual) were noted as major risks to girls’ wellbeing. Nearly all girl respondents in that country reported that parents or guardians regularly beat them as a form of discipline. Moreover, interviews and focus groups revealed that close relatives or teachers often perpetrated the sexual violence girls were subject to. In Tubmanburg, Bomi, one 14-year-old girl explained:

‘In school if a girl fails a particular subject and asks the teachers to help her, the teacher will want to sleep with her; she may accept to sleep with the teacher; then the teacher exploits the girl.’

Study respondents in Sri Lanka reported that adolescents’ physical and mental wellbeing, and particularly girls’ wellbeing, is closely linked to their protection and security. Influences on girls include the experiences of the adults in their lives who have lived through war and conflict. Often, these experiences continue to affect how those adults relate to their children even after the conflict has ended. In Gaza most respondents cited fear of sexual harassment as causing anxiety to girls and young women, especially those living in remote and/or border areas.

Other risks cited by respondents in all three countries included experiencing aggression or violence in the home, separation of parents and families, one or both parents migrating to find work, or a parent remarrying. All these circumstances can mean that young girls have to live with their extended family, which typically means taking on more domestic chores, loss of support for them to stay in school, and frequent exposure to harsh discipline and insults. In Gaza, for instance, girls faced the added vulnerability of being displaced from their homes, and reported distress at having to stay in mixed-sex shelters. (These were often schools or community centres temporarily repurposed to support displaced families whose homes were damaged by aerial bombing during the 2014 fighting.)

#### Coping responses

In all three countries, adolescent girls reported developing strategies to cope with psychosocial vulnerability. Individual-level coping strategies included drawing on ‘inner strength’ (girls in Sri Lanka); in Gaza, girls would read, draw, paint, write stories, use social media or simply daydream; while girls in all three countries mentioned turning to religion, embracing spirituality or consulting traditional healers.

‘The temple is the most important place for me. I like the calm environment it has’ (girl, 19, Diyagama, Sri Lanka).

‘I go to church. God saves us from troubles and all the sin we commit. God guards me and protects me from many things (girl, 15, Bomi, Liberia).

Across all three countries, girls cited support from the family (immediate nuclear family as well as extended family) as an important strategy in helping them cope with stresses. Aunts were often attributed with understanding girls more than their mothers did, as the aunts were often closer in age to the adolescent girls.

‘I’m asking God and my aunt to help me and to take care of my child while I’m in school’ (17-year-old adolescent mother, Bomi, Liberia).

However, we found that families can also undermine adolescent girls’ psychosocial wellbeing, whether due to violence, neglect and lack of privacy. For example, in Gaza, one adolescent lamented: *‘I don’t share problems with my mother. For one thing, she tells my father and the problem gets bigger!*’

Within the community, adolescent girls mentioned friends, teachers and staff working for service providers as being important in helping them cope with life’s challenges. Girls in Sri Lanka described how some teachers inspired them, gave them confidence and were willing to listen to their problems. In Sri Lanka and Gaza, girl respondents cited emotional support from a social worker or participation in psychosocial support programmes as helping them cope. Girls in all three countries described socialising and engaging in recreational activities as important coping strategies.

### Accessing formal service provision

In Sri Lanka and Gaza, there is a relatively established system of formal MHPSS service provision, whereas in Liberia such services are in their early stages. While the past decade has seen considerable progress in strengthening MHPSS systems in all three countries, our study respondents note that there are still limitations in how services and programmes meet girls’ specific age and gender needs. In Gaza, our findings suggest that despite MHPSS services being available, young people often do not access them due to cultural and other barriers. Whether in ‘normal’ every-day or crisis situations, services rarely target the needs of adolescents, with many programmes focusing on younger children.

Similarly, in Sri Lanka, although there are a wide range of MHPSS services, few are designed to deal specifically with adolescents, though the gender-based violence desks in hospitals (which offer psychosocial support to victims and survivors, children and adults) are a notable exception. Respondents in Sri Lanka also suggested the need to focus on social rather than medical interventions as a response to the actions of vulnerable adolescents, such as self-harm or suicide. Liberia also has some health and social support programmes targeting young people, but they vary widely in terms of effectiveness and impact.

In terms of gender-sensitive services and programmes, service user perceptions were mixed. Study respondents in Gaza reported that many service providers were enthusiastic and caring and wanted to help those accessing services. However, some (male) providers made judgements about the girls who sought such services and, in some cases, were not willing to treat the girl unless she had a chaperone (usually a family member). Not only did this undermine patient confidentiality, it also fuelled concerns around mental illness being a heritable issue, which could damage girls’ marriage prospects. According to one study respondent in Gaza (a caregiver): ‘*The general physician stopped following up my daughters, especially the older one, and he asked me to stop treating her because she is now a young lady, and continuing receiving mental health services will affect her reputation and she will be stigmatised forever… he said* “*It is enough. Don’t take her to any doctor. This will affect her if people know about her case*.*”’*

Drawing on respondents’ perspectives in all three countries, our findings highlight some critical gaps and disconnects in current MHPSS systems. First, according to study respondents, few activities focus on prevention or identifying groups of adolescents who may be at greater risk of mental ill-health. In Liberia, for instance, despite the existence of counsellors in schools, their role was limited to intervening only at times of crisis. ‘*The counsellors we have at school only counsel us when we fail—that’s when they call us to talk’* (16-year-old adolescent girl, Bomi, Liberia). Second, in all three countries, key informants noted that poor-quality and fragmented MHPSS services remain a critical challenge to providing a unified response. Thirdly, respondents noted the need for greater strategic direction for MHPSS services, which are hampered by a short-term approach and limited (or a total lack of) follow-up mechanisms. Fourth, lack of evidence-based practice was mentioned by many respondents. Finally, in all three contexts, lack of essential resources (financial and human) remains an important constraint according to all study respondents.

A theme that was brought up by the majority of study respondents was the negative attitudes people displayed towards using mental health services in general and in particular to adolescent girls using such services. In all three countries, people experiencing mental health problems can face substantial stigma and discrimination. In Gaza, for example, most study participants felt that although various efforts had been made to reduce stigma (e.g. through carrying out awareness-raising programmes), they have largely proved ineffective; the result is, according to study respondents, that many people delay seeking help from those services and often do so only after using traditional healers.

Adolescent girls’ psychosocial wellbeing covers many dimensions and sectors, yet the provision of services remains siloed and constrained by weak coordination. In Gaza, respondents were concerned that there was very limited coordination between psychosocial and mental health service providers and programmes, minimal exchange of information and feedback, and hardly any systematic follow-up of referred cases. In Liberia, respondents also noted that there were few synergies across sectors providing specific MHPSS services or programmes. Moreover, despite mental health professionals having received training in child and adolescent services, they have tended to focus their activities on adults and individuals with serious mental illness. In Sri Lanka, vertical integration between key sectors (health, education and child protection), from community-based services to district- and national-level mechanisms (although there remains a strong focus on adolescent girls’ protection and medical needs) was identified in policies and approaches by study respondents. However, on the ground, service providers noted that collaboration across sectors is generally low. The situation is similar for horizontal linkages—while there are many non-government organisations (NGOs) working in different sectors, most, according to study respondents, work independently at local level.

## Discussion

Overall, four broad insights emerged from our study that also contribute to literature on the social determinants of health (e.g. Braveman and Gottlieb 2014, Viner et al. 2012). Firstly, it is vital that social determinants are located within specific political economy understandings to take into account the complex and multi-layered challenges that affect access to all services including mental health and psychosocial support services. In our case study countries the political economy is characterised by fragility in terms of governance, the rule of law and institutional capacities and coordination. This fragility will clearly impact the kind, level, comprehensiveness and quality of service provision as well as the extent to which service are accessible and affordable, and particularly for adolescent girls.

While all three case study countries are classified as post-conflict, we found that the political economy dynamics vary significantly, with important implications in terms of psychosocial vulnerabilities and the service environment. In Sri Lanka, poverty emerged as the dominant driver of psychosocial vulnerability among adolescent girls, although the conflict persists as a shadow in their lives. By contrast, in Gaza, conflict (or the risk thereof) due to the Israeli blockade and periodic bombings is ever-present for everyone, but with particular challenges for adolescent girls. In Liberia, despite the fact that the conflict formally ended >10 years ago, levels of sexual violence and abuse of women remain among the highest in the world.^5^ This reflects what others have termed ‘hyper-masculinity’, whereby social norms around masculinity normalise and reinforce practices of sexual and gender-based violence (SGBV) rather than seeking to reframe acceptable male attitudes and behaviours as part of the broader peace-building process (see e.g. Barker and Ricardo 2005, Domingo et al. 2013, Fleming et al. 2015, Wright 2014).

Secondly, although attention to social and gendered norms within the social determinants of health literature is growing, it remains too often cursory and analysts fail to disentangle the wide-ranging consequences of social and gendered norms, both on health and wider psychosocial wellbeing. Integrating such a perspective is thus critical and what the current study also strives to do.

Thirdly, and linked to the second broad insight, our study highlights the particular psychosocial vulnerabilities adolescent girls face and how they are closely linked to and intertwined with a range of gendered social norms that begin to bear heavily on girls’ lives as they enter adolescence. Thus informed by our conceptual framework, which outlines adolescent girls’ pathways to psychosocial wellbeing, our study suggests that norms around mobility, marriage, sexuality, education, time use and emotional life underlie and explain much of the behaviour of adolescent girls, including how they perceive and experience mental health and psychosocial distress as well the forms of support, both informal and informal, both available to them and which they are able to access. Yet the role that gendered social norms can play in influencing accessibility and update of services is too often neglected in health systems thinking in many developing country contexts. In Sri Lanka, for instance, barriers to psychosocial service uptake for girls include the stigma associated with admitting to psychosocial problems and the fact that a girl cannot voice questions and interest in adolescence, since it is highly associated with taboos around sex and sexuality which are in turn guided by underlying gendered social norms. Similarly, gendered social norms also continue to hamper girls’ ability to take up MHPSS services. Among Muslim communities in Sri Lanka religious institutions and leaders typically mediate in cases that involve sexual abuse and early pregnancy, often ‘resolving’ the matter by ordering the perpetrator to marry the girl. Hence norms around marriage/early marriage in this case override possibilities of girls accessing MHPSS should they be available. Given this context, services were dedicated to addressing ‘risks’ and providing ‘protection’ for girls, framing their activities around regulating girls’ ‘questionable or immoral behaviour’ rather than an overriding concern to improve girls’ psychosocial wellbeing.

In Gaza, gendered social norms which restrict girls’ mobility, largely because of fear that this mobility would damage ‘family honour’ often result in them facing social isolation which can in turn fuel further mental health and psychosocial distress. Similarly the stigma related to MHPSS service uptake in Gaza also inhibits them seeking support since this will likely harm girls’ marriage prospects, and given that marriage is one of the most significant life stages in a person’s life, and underlies much of the social order, this becomes more important than seeking on MHPSS services.

And in Liberia, aggressive masculinities—a legacy of the conflict—and the normalisation of sexual violence, again a key underlying gendered social norm, remain major threats to girls’ physical and psychosocial wellbeing, for which services and support remain highly inadequate.

Fourthly, although psychosocial wellbeing is arguably subsumed within the social determinants of health framework, our findings highlight that more explicit attention must be paid to psychosocial wellbeing and mental health in conflict-affected contexts, and that these dimensions should be accorded equal weighting to adolescents’ physical health concerns.

Finally our findings suggest that unless these insights are well understood, efforts to strengthen the human resources needed to support adolescent girls’ health and wellbeing may prove ineffective, and may even inadvertently uphold discriminatory gendered social norms that often underpin local health practices.

## Conclusions

Reflecting on the implications of our findings for future policy and practice, we highlight three broad cross-cutting areas that can also be modified for other contexts.

First, our study findings highlight that a range of actions are needed to (1) address the factors that contribute to girls’ psychosocial vulnerabilities, (2) boost girls’ coping strategies, and (3) mitigate against negative coping responses. Such actions include taking a proactive approach to identifying vulnerable groups (including adolescent girls and those at greatest risk of SGBV) and tailoring services and activities to meet their needs. Low-resource measures for mitigating the risk of psychosocial ill-being in fragile contexts could include: encouraging the use of (peer and other) social networks to support adolescents, providing safe spaces for girls, and providing training (to adolescents and their caregivers) on positive coping strategies, including ways to reduce stress. Building on and working with supportive teachers, as well as harnessing the role of the education sector more broadly to help develop adolescents’ self-esteem and self-efficacy, also emerged as a critical area for investment. Finally, the generalised stigma that hinders access to psychosocial services needs to be addressed, with substantial efforts for outreach through the media and community mobilisation.

Secondly, our findings also indicate that building the capacities of service providers is critical to stimulate an improved service environment. Areas for capacity-building include: early diagnosis of mental health illnesses, provision of SGBV-related services, and treating substance abuse. The service environment, however, should not be limited to these more medicalised approaches; it should include wide-ranging activities, from promoting girls’ participation in social activities, to providing spaces for creative expression, as well as providing safe spaces for girls to play and enjoy leisure time. Finally, there is a need for more investment to make services proactive and integrated. In the education sector, for instance, the quality of school-based psychosocial support, counselling services and protocols need to be improved; similarly, in the justice sector, the police need to receive adequate training on SGBV and how to support adolescent girls who are victims or survivors.

A third set of actions concerns the need to enhance policy strategies, and to regulate and coordinate actors providing MHPSS services at different levels (community, sub-national and national). There is also a need to strengthen national institutions and ministries so that they become recognised as legitimate regulators of psychosocial services, and can provide, among other things, improved licensing and accreditation processes. Finally, our findings underscore the need to provide more evidence to inform programming, and particularly the need for more robust and disaggregated data to reflect the realities of adolescents’ lives.

In sum, our findings highlight the importance of recognising adolescents’ psychological needs and their gendered patterning, and working to ensure that their needs are integrated into programme design and implementation. Such efforts need to simultaneously tackle the underlying gendered social norms affecting attitudes and behaviours both around psychosocial and mental health service uptake and service provision. This is particularly pertinent in fragile and post-conflict settings if women and girls are to become active agents in change processes, including those related to peace processes and conflict resolution.

## Ethical approval

Ethical approval was sought and obtained through the ODI ethics review board. In addition, the study was also cleared by national research ethics boards in the study countries, i.e. in Liberia, The University of Liberia, the Pacific Institute for Research and Evaluation (PIRE) approved the research, in Gaza the study was reviewed by the Helsinki Framework ethics board, and in Sri Lanka the study was reviewed by a group of practitioners and researchers with relevant expertise.

## Footnotes

1
http://www.who.int/social_determinants/en/

2 For details of the literature review please see Stavropoulou and Samuels, 2015.

3 In Gaza only one round of data collection took place since it was added to the study at the time when it thought the team would not be able to continue fieldwork in Liberia due to Ebola; in fact the team was able to carry out a shorter second round of data collection in Liberal post the height of the epidemic.

4 Transactional sex is widespread throughout sub-Saharan Africa and there is a large body of literature on this. For a recent overview article please see Stoebenau et al., 2016.

5 Rape of girls and women persists and accounts for over one-third of sexual violence cases. Victims are mostly young people (10-19), with male perpetrators mostly known to the victim and largely (almost 40%) between the ages of 20-39 (GoL, 2011).
